# From salty to thriving: plant growth promoting bacteria as nature’s allies in overcoming salinity stress in plants

**DOI:** 10.3389/fmicb.2023.1169809

**Published:** 2023-06-23

**Authors:** Mu Peng, Zhihui Jiang, Fangzhen Zhou, Zhiyong Wang

**Affiliations:** ^1^Hubei Key Laboratory of Biological Resources Protection and Utilization, Hubei Minzu University, Enshi, China; ^2^College of Biological and Food Engineering, Hubei Minzu University, Enshi, China

**Keywords:** plant growth-promoting bacteria, salinity stress, plant microbiome, Type VI secretion system, plant nutrients, phytohormones, osmolytes

## Abstract

Soil salinity is one of the main problems that affects global crop yield. Researchers have attempted to alleviate the effects of salt stress on plant growth using a variety of approaches, including genetic modification of salt-tolerant plants, screening the higher salt-tolerant genotypes, and the inoculation of beneficial plant microbiome, such as plant growth-promoting bacteria (PGPB). PGPB mainly exists in the rhizosphere soil, plant tissues and on the surfaces of leaves or stems, and can promote plant growth and increase plant tolerance to abiotic stress. Many halophytes recruit salt-resistant microorganisms, and therefore endophytic bacteria isolated from halophytes can help enhance plant stress responses. Beneficial plant-microbe interactions are widespread in nature, and microbial communities provide an opportunity to understand these beneficial interactions. In this study, we provide a brief overview of the current state of plant microbiomes and give particular emphasis on its influence factors and discuss various mechanisms used by PGPB in alleviating salt stress for plants. Then, we also describe the relationship between bacterial Type VI secretion system and plant growth promotion.

## 1. Introduction

Plants have relationships with various members of an ecosystem and can grow well in their natural environments. Plant microbiome refers to all microorganisms that survive in a specific environmental niche at a given time, and is one of the most important organisms that can have beneficial effects for plants, such as improving nutrient uptake, disease resistance, and stress tolerance (Santoyo et al., 2016; Trivedi et al., 2022; Xiong and Lu, 2022). Microbial communities can improve plant tolerance to biotic and abiotic stresses, and increase plant growth and yield by improving bioavailability for transporting nutrients from the soil. Microbial communities vary across different environments and are generally thought to be tissue-specific; they mainly colonize in root and tissues of plants or around roots in the soil. Bacteria that colonize and survive in the host plant are called endophytes (Bohra et al., 2022). However, the beneficial effects of endophytic bacteria on host plants are generally greater than those of plant growth promoting rhizosphere (PGPR), and these effects may be enhanced especially when plants are under stress (Miliute et al., 2015; Malik and Arora, 2022).

Plants start to establish their microbial community system, known as the seed-borne microbiome that is transmitted from the seed to the plant (Nelson, 2018; Abdelfattah et al., 2022), from the seed stage. The interplay between plants and their endophytes during the plant’s entire life cycle may have profound impacts on plant ecology, health, and productivity. The niche differentiation of microbial communities at the soil-root interface has attracted the most attention, due to different plant compartments provide different ecological niches for microbes living in them (Edwards et al., 2015). Plant roots (in the rhizosphere) act as a bridge connecting plants and soil microbes and secrete many photosynthates to the surrounding soil environment, providing a very attractive and nutrient-rich niche for microbes and making the rhizosphere have the highest microbial diversity (Singh and Mukerji, 2006).

Salt stress is one of the main factors limiting agricultural productivity and causing a significant loss of global crop yields (Ha-Tran et al., 2021). In order to cope with harsh environments, many strategies have been implemented, such as plant breeding, plant genetic engineering and climate-smart agriculture (Upadhyaya et al., 2021). Plant growth promoting bacteria (PGPB) have been shown to effectively improve plant tolerance to abiotic stress and increase crop yield, making them promising biofertilizers (Das et al., 2022; Zilaie et al., 2022). However, its unstable growth-promoting effect and unclear mechanism seriously hinder the use of microbial agents. In this review, we provide a brief overview of the current state of plant microbiomes and give particular emphasis on its influence factors and discuss various mechanisms used by PGPB in alleviating salt stress for plants. Then, we also describe the relationship between bacterial Type VI secretion system and plant growth promotion.

## 2. Effects of salt stress on plant growth

Soil salinity is one of the main abiotic stresses that limit plant growth and yield. Plants under salt stress have significantly reduced productivity, and the extent of these effects depends on salt content, plant type, and the stage of plant growth and development. Many studies have reported that high levels of soil salinity can inhibit see germination, significantly reduce root and shoot growth, as well as decrease plant photosynthesis, stomatal conductance, chlorophyll content, and mineral uptake (Laghmouchi et al., 2017; Sánchez-García et al., 2017). Currently, the mechanisms by which salt stress affects plant growth mainly include disturbances in plant hormone balance, changes in protein metabolism, inhibition of enzyme activity involved in nucleic acid metabolism, and nutrient uptake disorders, which are caused by osmotic effects and salt ion toxicity (Zhu, 2001; Amirjani, 2011). In addition, studies have also found that salt can inhibit cell membrane and cell wall maturation (Ismail and Horie, 2017).

The high salt concentration around the plant roots increases osmotic stress, which in turn leads to ion toxicity. Osmotic stress mainly affects water and nutrient uptake, seed germination, cell elongation, leaf development, lateral bud development, photosynthetic rate, transport from root to stem, and the supply of carbohydrates to meristematic tissues (Van Zelm et al., 2020). The ion toxicity of Na^+^ and Cl^−^ hinders the absorption of nutrients such as Ca^+^ and K^+^, causing an imbalance in plant nutrition (Acosta-Motos et al., 2017). Soil salinity prevents the absorption of Ca^+^ by plants, affecting the growth of roots, root tips, and root hairs, reducing the areas where rhizobia can invade and the development of nodules (Bouhmouch et al., 2005). In addition, the increase in Na^+^ and Cl^−^ content reduces the absorption and utilization of some elements (N, P, K, Mg) by plants. The imbalance of minerals usually changes the structure and chemical composition of the lipid bilayer of the membrane, and controls the selective transport of solutes and affects the ability of ions to transport inward, causing solute leakage and forming super-osmotic phenomena (Lodhi et al., 2009).

Ion toxicity also destroys the photosynthetic apparatus by blocking the photosystem II reaction center, oxygen-evolving complex, and electron transport chain, thereby inhibiting photosynthesis (Kan et al., 2017). The accumulation of a large amount of Na^+^ in plant tissues suppresses photosynthesis, resulting in the accumulation of reactive oxygen species, which have many adverse effects on plants, such as accelerating toxic reactions, DNA mutations, protein degradation, and membrane damage (Islam et al., 2015). Salt also has a negative effect on plant height and root length, causing stomatal closure, increasing leaf temperature, and reducing elongation (Yavaş and Hussain, 2022). As salt concentration increases, plant height and root length tend to decrease, and these negative results are related to changes in osmotic potential and the decreased ability of rice to absorb water and nutrients (Gain et al., 2004). Furthermore, stomatal closure can increase plant CO_2_ deficiency, leading to a decrease in enzyme activity in the Calvin cycle (Ashraf et al., 2020). In addition to the effects on plant organs and structures mentioned above, root zone salinization also hinders the developmental stages of individual plants. Overall, from seed germination to seed formation stages, salt has a serious impact on the physiological and biochemical activities of plants.

## 3. Plant microbiome

In natural ecosystems, organisms do not live alone, but in close association with a wide variety of microorganisms. In nature, plants do not grow like axenic organisms, but host a diverse microbial community named the plant microbiome (Müller et al., 2016). The plant microbiome plays an essential role in plant growth, improving the bioavailability of nutrients and increasing the host’s tolerance to biotic and abiotic stresses. In turn, host plants provide habitat and nutrients for microbes (Trivedi et al., 2020). In fact, microbial communities exist in almost all plant tissues, such as the soil-root interface (rhizosphere), the interior of plant tissues (endophyte), and the air-plant interface (phyllosphere) (de Medeiros Azevedo et al., 2021). All these microenvironments provide specific biotic and abiotic conditions for microbial communities to survive. Understanding plant-microbe interactions not only provides a better understanding of the role of microbes in plant growth and development, but also allows the use of their relationships for phytoremediation, sustainable crop production, and secondary metabolite production. However, it is important to note that the plant microbiome can be influenced by various environmental factors, host and other biotic and abiotic conditions. The plant microbiome contains mixed populations of taxa from diverse phyla, in which each member adapts to the host microenvironment in some way and co-exists with other members. Nonetheless, determining plant-microbiome interactions can be challenging due to the complexity of microbial community assembly mechanisms.

### 3.1. Phylogenetic structure of the plant microbiome

Healthy plants host taxonomically diverse microbial communities (Trivedi et al., 2020). Terrestrial plants are colonized by a huge variety of microorganisms that affect plant health and growth in beneficial, harmful, or neutral ways. Previous studies have confirmed that the host-microbial communities are not generally random assemblages, but show defined phylogenetic structures, and only a few major groups constitute the plant microbial communities with high abundance, including Proteobacteria, Actinobacteria and Bacteroidetes (Müller et al., 2016).

Many studies aimed to identify the core microbiome by phylogenetically distinct plant species or compartments. Yeoh et al. (2016) determined the core root microbes of sugarcane, including Proteobacteria, Actinobacteria, Bacteroidetes, Chloroflexi, and Firmicutes, which were found to overlap with those of *Arabidopsis thaliana,* suggesting that some bacterial families may have a long-term association with plants (Yeoh et al., 2016). The study of core phyllosphere microbiomes of *Arabidopsis*, soybean and clover found that a high consistency of communities in leaves of these different plants (Delmotte et al., 2009). Proteobacteria, Actinobacteria and Bacteroidetes were the core phyllosphere microbiota in these three plants (Delmotte et al., 2009). Kembel et al. (2014) tested the relationships between the phyllosphere bacterial biodiversity and the functional traits, taxonomy and phylogeny of their plant host, and confirmed that the structure of phyllosphere bacterial community was highly correlated with the evolutionary relatedness of hosts and a series of plant functional traits related to host ecological strategies (Kembel et al., 2014). Similarly, Leff et al. (2015) observed the bacterial community composition is highly variable, but this variability is predictable and dependent on plant compartments (Leff et al., 2015). This work highlights the importance of considering plant spatial structure when studying plant-associated microbial communities and their effects on plant hosts.

The plant microbiome interacts with its host in a variety of manners and affect host’s growth. However, relatively little information is available on the composition and diversity of bacterial communities in different aboveground plant organs, especially those associated with flowers. Zarraonaindia et al. (2015) revealed some unique plant-associated microbiota by comparing the bacterial communities in grapevine leaves and flowers; the flower-associated microbiota almost entirely composed of Proteobacteria (Zarraonaindia et al., 2015). Junker and Keller (2015) determined that the leaves microbiome of *Metrosideros polymorpha* hosts a unique indicator community composed of relatively abundant bacterial groups; these indicator communities are accompanied by a large number of ubiquitous or rare bacteria with lower abundances.

### 3.2. Plant-associated microbiome assembly and driving factors

Microorganisms colonizing the host plant benefit from nutrients provided by the plant and form taxonomically consistent community patterns (Müller et al., 2016). This mechanism consists of two aspects: on the one hand, plants provide unoccupied niches for invading microorganisms, which are able to take advantage of the provided niche or nutrients, resulting in stochastic colonization events; on the other hand, plant-microbe coevolution might provide the basis for plant-driven selection processes, leading to key species that actively recruit microbial members or at least provide functions to plant hosts, which contribute to the shaping of the ultimate microbial community during plant development (Müller et al., 2016).

Plants act as environmental filters to shape the microbial communities, and there are many studies about the effects of microbial communities on their hosts (Adair and Douglas, 2017). However, the study of the mechanisms underlying the assembly of the plant microbiome is still in its infancy. Based on microbial communities and genetic evidence, Reinhold-Hurek et al. (2015) proposed a enrichment model of root-related microbiomes, explaining the reasons for the decreased diversity and increased specificity of microbial communities from the bulk soil to the root interior (Reinhold-Hurek et al., 2015). Soils provide a highly diverse microbiome that is influenced by soil physicochemical factors, environmental factors, and vegetation types. This process is mainly divided into three steps. In the first step, in the rhizosphere, the community may be refined due to the influence of roots, such as carbon source, oxygen, pH or nutrient consumption. In the second step, the community shows more obvious refinement in close contact with the host on the root surface, and plant genotype has a significant influence. Biofilm formation or specific adhesion mechanisms may be involved in this enrichment step. Third, in the least complex community, Proteobacteria is a fast-growing bacterium with high metabolic activity, which dominates the endosphere bacterial community. For endophytic bacteria, specific bacterial traits are required to colonize in plant tissues, such as community sensing, plant polysaccharide degradation or resistance to reactive oxygen species. Compared with other plant compartments, host genotype may have the greatest influence on community structure in this habitat.

The establishment of plant microbiome is a dynamic process, reflecting the changes of community composition over time in response to environmental changes. Among them, environmental conditions, root exudates, microbe-microbe and plant-microbe interactions, and various stages of plant development all affect the assembly of microbial communities (Huang et al., 2014). It is not clear whether bacteria establish a founding community early in plant development, and then constantly colonize in the new habitat by clonal propagation (Müller et al., 2016). Therefore, it is very difficult to understand a system as complex as the plant microbiome and to distinguish between co-evolving interactions and stochastic processes.

So far, little is known about the source of microbial communities and how plants shape their specific microbial communities. Although the initial bacterial communities are similar to their respective seed banks, they become more specific and more diverse as plants grow and develop (Chaparro et al., 2014; Müller et al., 2016). Hardoim et al. (2012) found that about 45% of the bacterial communities in rice progeny seeds were similar to the parental plant, indicating that the rice microbiome could be directly obtained from seeds and transmitted in the form of endophyte (Hardoim et al., 2012). The diversity and dynamics of seed microbiota represent the culmination of a complex process of microbial interactions mediated by the plant throughout its life cycle (Nelson, 2018). Microbes associated with endosperm are more likely to vertically spread, while those associated with seed coat are more likely to horizontally transmit (Barret et al., 2016).

Soil represents an extremely rich microbial pool on Earth, serving as a major seed bank for the microbiota of the rhizosphere and root, and a driving force for community formation (Müller et al., 2016). In many studies, it has been confirmed that soil type has a significant impact on rhizosphere microbial communities (Gottel et al., 2011; Lundberg et al., 2012; Edwards et al., 2015). In addition, under the same environmental and soil conditions, plant species and genotype are one of the driving factors for the structural and functional diversity of plant microbiomes (Matthews et al., 2019). The functional diversity of the microbiome is also affected by plant varieties or cultivars. Even the sister lines of the same hybrid had different root-associated diazotrophs in rice (Knauth et al., 2005). Moreover, the influence of plants on microbial communities seems to be heritable, reflected in the finding that the gene expression of the microbiota in interspecific hybrid rice showed the intermediate level between the parental species (Knauth et al., 2005). These studies suggest that plants act as filters for their microbiome. However, quantitative analysis of microbial community composition between ecotypes or plant varieties showed that differences in microbial diversity related to genotypes were relatively small compared with environmental factors (Edwards et al., 2015).

Finally, microorganisms themselves can also influence the establishment of microbial communities, which can be mediated via either directly at the microbial-microbial interaction or indirectly through host-microbial interactions. This feature has been confirmed in many studies. For example, Chapelle et al. (2016) analyzed the rhizosphere microbiome of sugar beet seedlings, and found that invasive pathogenic fungi could induce stress responses in rhizobacterial communities, leading to shifts in microbial community composition (Chapelle et al., 2016). Han et al. (2020) determined that some rhizosphere microbes in nodules were related to the composition of rhizobia, and confirmed that bacterial communities played a crucial role in the interaction between soybean rhizobia and host (Han et al., 2020). Niu et al. (2017) assembled a simple and representative synthetic bacterial model and studied the community assembly process of maize seedlings. The abundance of representative strains was monitored by selective culture-dependent method, and the role of each strain in community assembly was studied. When *Enterobacter cloacae* was removed, the community structure was completely lossed, indicating that *E. cloacae* plays the role of key species in this model ecosystem (Niu et al., 2017). In summary, current studies indicate that microbe-microbe-host interactions are one of the factors affecting the assembly of the plant microbiome.

## 4. PGPB mediated salinity-tolerance in plants

**Figure 1:**
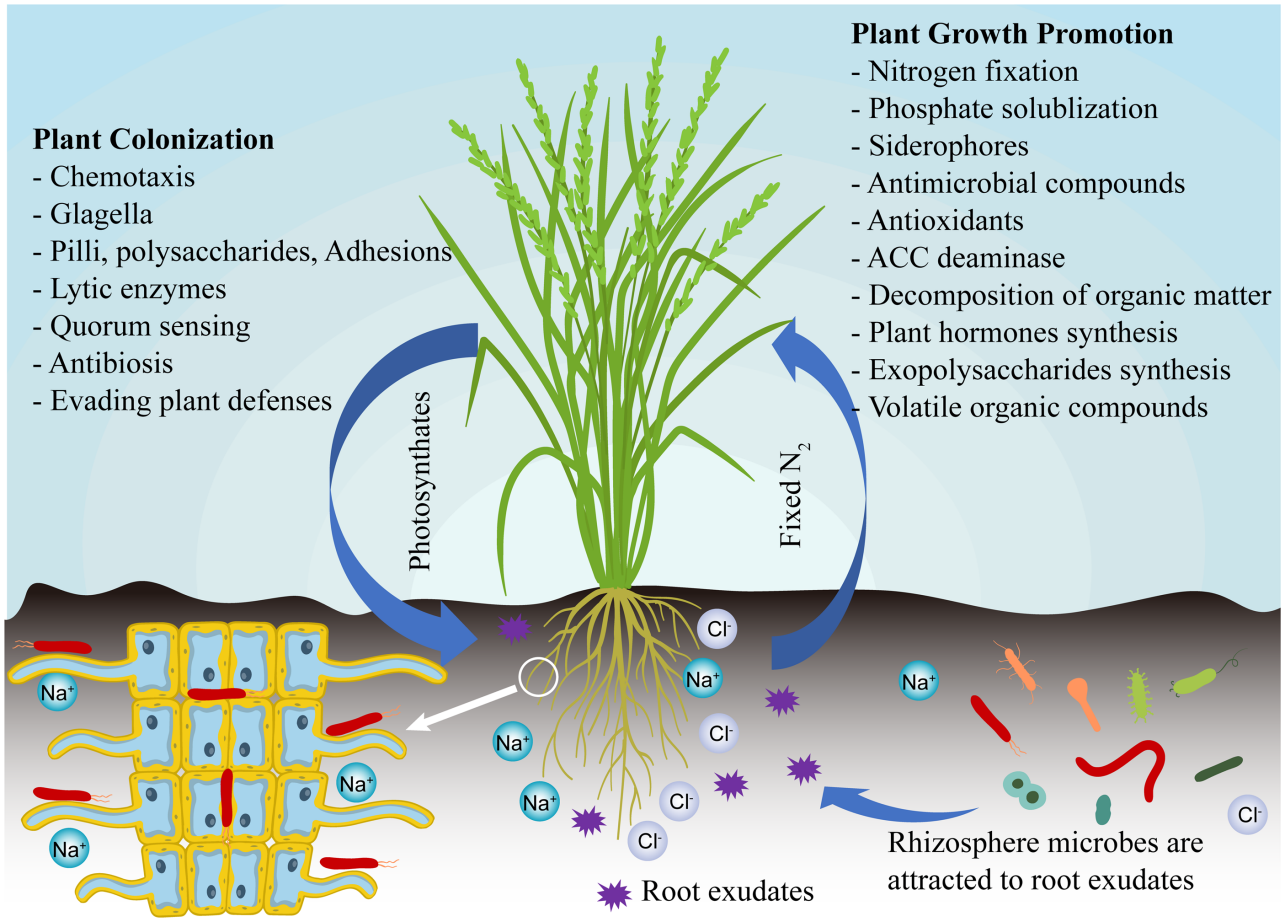
PGPB mediate direct or indirect mechanism to improve salt tolerance in plants.

Plant growth-promoting bacteria (PGPB) is a kind of beneficial free-living bacteria in soil or colonized in plant tissue, which can promote plant growth and nutrient uptake, improve plant disease resistance, and suppress harmful microbes (Hoque et al., 2022; Khatoon et al., 2022). The most important thing is that PGPB is safe for the environment, humans and animals (Khabbaz et al., 2019). PGPB includes microorganisms isolated from different ecosystems such as soil, plants, and oceans, which can colonize the internal plant tissues and have several beneficial effects on the host directly or indirectly under salinity stress (Figure 1). Although thousands of PGPB species have been isolated over the past few decades, the mechanism of plant-growth promotion is still uncertain. Once successfully colonized in the rhizosphere as well as plant tissues, PGPB can help to facilitate plant growth through various mechanisms, such as regulating plant hormones and nutrient acquisition, and reducing ethylene production (Afzal et al., 2019). PGPB can also indirectly control plant health by suppressing infectious diseases (e.g., production of antibiotics and lyases, inhibition of nutrient uptake by pathogens, and activation of plant defense mechanisms), thereby protecting plants from pathogen attack (Miliute et al., 2015). Because of their extensive properties in maintaining crop health, as well as their environmentally friendly nature, PGPB have become an important component of sustainable agricultural development (Das et al., 2022).

PGPB includes two groups of bacteria, namely, rhizospheric bacteria near plant roots and endophytic bacteria in plant tissues. In most cases, rhizospheric and endophytic PGPB use similar mechanisms to promote plant growth, and the main difference is that endophytic PGPB once colonizes the tissues of the host plant and is no longer affected by the vagaries of soil environmental conditions (Akhtar et al., 2022). These changing soil conditions may inhibit the function and proliferation of PGPB in the rhizosphere, and other soil bacteria may compete for the same invasive sites in the root surface of host plants. PGPB have been found as a promising way of boosting the yield and growth of salinity-stressed plants (Table 1).

**Table 1:** Impact of PGPB in alleviating salinity stress in plants.

Plant species	PGPB species	Effect or mechanism	References
Canola	*Enterobacter* sp. S16-3 *Pseudomonas* sp. C16-2O	Production and accumulation of reactive oxygen species	Neshat et al. (2022)
*Zea mays* L.	*Metabacillus dongyingensis* BY2G20	cation-proton antiporters, cation transporters, osmoprotectant synthesis and transport, H^+^-transporting F1F0-ATPase, indole-3-acetic acid production	Yin et al. (2022)
*Coriandrum sativum*	*Pseudomonas pseudoalcaligenes* KB-10 *P. putida* (KB-25)	Increase relative water content, concentrations of photosynthetic pigments, peroxidase activity, total biomass, salt tolerance index, and reduced salt-induced total phenolic contents	Al-Garni et al. (2019)
*Lotus japonicus cv. Gifu*	*Bacillus amyloliquefaciens* RHF6	Induce a plant antioxidant response, secrete the osmoprotectant proline and reduce ethylene level via the enzymatic ACC deaminase activity	Castaldi et al. (2023)
*Phaseolus vulgaris* L.	*Bacillus proteolyticus* Cyn1 *and B. safensis* Cyn2	ACC deaminase production, phosphate solubilization, and catalase enzyme secretion	Meza et al. (2022)
Iranian licorice (*Glycyrrhiza glabra* L.)	*Azotobacter* sp.	Increase polyphenol oxidase, peroxidase, and phenylalanine ammonia-lyase activity	Mousavi et al. (2022)
Spinach and Soybean	*Stenotrophomonas* sp.	Accumulate osmolytes, increase enzymatic and non-enzymatic antioxidants	Nigam et al. (2022)
Tomato	*Pseudomonas* sp. UW4	Production of ACC deaminase and trehalose	Orozco-Mosqueda et al. (2019)
Soybean (*Glycine max* (L.) Merr; Leguminosae)	*Bacillus cereus* (B1), *B. megaterium* (B2), *Trichoderma longibrachiatum* (F1) and *T. simmonsii* (F2)	Improve germination, seedling growth and potassium uptake	Bakhshandeh et al. (2020)
Rice (*Oryza sativa*)	*Bacillus* sp., *Exiguobacterium* sp., *Lysinibacillus* sp., *Stenotrophomonas* sp., *Microbacterium* sp., and *Achromobacter* sp.	Enhance total chlorophyll, proline, total phenol, and oxidative damage such as electrolyte leakage and membrane stability index	Prittesh et al. (2020)
Rice (*Oryza sativa*)	*Bacillus pumilus* strain JPVS11	Promote photosynthetic pigments, proline and antioxidant production	Kumar et al. (2021)
Rice (*Oryza sativa*)	*Brevibacterium linens* RS16	Increase in total proline and glycine betaine accumulation	Ahmed et al. (2021)
*Amaranthus Viridis*	*Bacillus safensis* *Bacillus haynesii*	Produce gibberellic acid, indole-3-acetic acid, hydrogen cyanide, ammonia, 1-amino cyclopropane-1-carboxylic acid deaminase, exopolysaccharides, protease, chitinase, amylase, cellulase, and solubilized minerals such as phosphorous, zinc, and potassium	Patel et al. (2023)
Wheat	*Bacillus megaterium*, *B. tequilensis*, and *Pseudomonas putida*	Decrease concentration of malondialdehyde and hydrogen peroxide Reduce electrolytic leakage and enhance enzymatic activity for the scavenging of reactive oxygen species (ROS); Increase the production of proline and total soluble sugar	Haroon et al. (2021)
Pea (*Pisum sativum*)	*Acinetobacter bereziniae* IG2, *Enterobacter ludwigii* IG 10, *Alcaligenes faecalis* IG 27	Increase chlorophyll content, proline content, total soluble sugar, electrolyte leakage, and activities of antioxidant enzymes; Decrease the levels of electrolyte leakage and H_2_O_2_ content	Sapre et al. (2022)
*Cucumis sativus* L	*Serratia fonticola* S1T1	Decrease in malondialdehyde content, H_2_O_2_ content and superoxide anion, increase in antioxidant enzymes such as catalase and superoxide dismutase; up-regulate the transcript accumulation of ion transporter genes *HKT1*, *NHX* and *SOS1*	Moon et al. (2023)
*Metasequoia glyptostroboides*	*Bacillus paramycoides* JYZ-SD5	Increase the activities of superoxide dismutase (SOD), peroxidase (POD), Na^+^–K^+^-ATPase and Ca^2+^–Mg^2+^-ATPase	Kong et al. (2022)
Oat (*Avena sativa* L.)	*Bacillus sp.* LrM2	Inhibit the accumulation of H_2_O_2_ and malondialdehyde; enhance the activities of catalase, ascorbate peroxidase, glutathione reductase, and monodehydroascorbate reductase were enhanced, and the levels of the non–enzymatic antioxidants, ascorbate and glutathione	Zhang et al. (2022)
*Lathyrus cicera*	*Rhizobium laguerreae* *Rhizophagus irregularis* *Bacillus subtilus, Bacillus simplex and Bacillus megaterium*	Upregulate the expression of two marker genes (*LcHKT1* and *LcNHX7*) related to salinity tolerance	Gritli et al. (2022)
Soybean (*Glycine max*)	*Enterobacter* Delta PSK and *Bradyrhizobium japonicum*	Decrease electrolyte leakage, the amounts of malondialdehyde and hydrogen peroxide	Agha et al. (2023)
Sugarcane (*Saccharum* sp. Hybrids)	*Acinetobacter* sp. RSC9	Produce IAA, solubilize phosphate, potassium and zinc and fix atmospheric nitrogen	Patel et al. (2022)
Sweet potato (*Ipomoea batatas* L.)	*Klebsiella sp.* San01	Enhance 2,2-diphenyl-1-picrylhydrazyl radical scavenging ability and elevate activities of ascorbate peroxidase and superoxide dismutase	Li et al. (2022)
*Musa acuminata* cv. Berangan	*Bacillus* sp., *Pseudomonas* sp.	Enhance levels of plant chlorophyll, carotenoid and proline, reduce MDA content, ROS and electrolyte leakage	Kaleh et al. (2022)
Rice (*Oryza sativa*)	*Bacillus megaterium*	Affect K^+^ uptake not by solubilizing it but changing K^+^ transporters expression	Romero-Munar and Aroca (2023)
Lima bean (*Phaseolus lunatus*)	*Bradyrhizobium* and *Azospirillum baldaniorum*	Increase nitrogen compounds (nitrate, free ammonia, free amino acids, proline, and soluble protein); increase in sodium and the highest potassium content values, nitrogen derived from the atmosphere, and nitrogen fixation efficiency	de Oliveira Lopes et al. (2022)

### 4.1. Availability of plant nutrients

**Figure 2:**
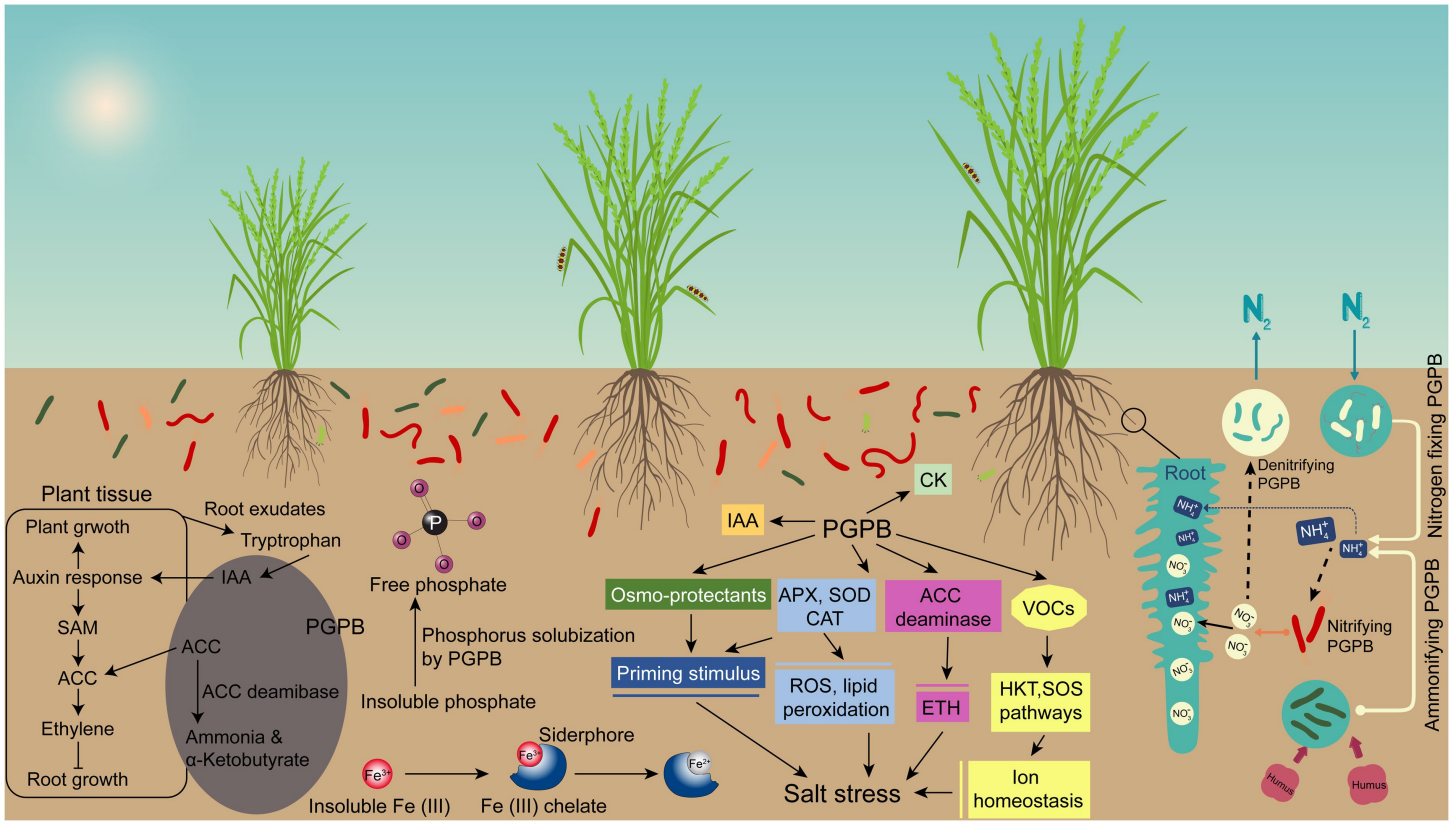
The potential mechanism of bacteria-mediated plant growth promotion under salt stress. Plant Growth Promoting Bacteria (PGPB) serve as a biofertilizer promote plant growth by enhancing nutrient availability, regulating plant hormones, and increasing plant stress tolerance. They increase the concentrations of growth hormones, such as gibberellins, cytokinins, while decreasing ethylene levels through ACC deaminase. Moreover, PGPB also produce volatile organic compounds (VOCs) that induce disease resistance and abiotic stress tolerance. In addition, PGPB alleviates stress by augmenting extracellular polysaccharides, osmoregulants, and antioxidants, thereby reducing reactive oxygen species and oxidative stress.

Many agricultural soils are of poor quality due to deficiency of one or more plant nutrients, so plant growth in this condition is suboptimal. In order to solve this problem and achieve higher crop yields, cultivators increasingly rely heavily on inorganic chemical-based fertilizers of nitrogen and phosphorus. The production of chemical fertilizers is not only costly, but also depletes non-renewable resources, such as oil and gas, and causes harmful effects to humans and the environment (Shirokov and Tikhnenko, 2021). It is clearly beneficial and sustainable if effective biological means of providing nitrogen and phosphorus to plants could be used to replace part of the chemical nitrogen and phosphorus usage (Glick, 2012). PGPB can help host plants to uptake nutrients, including nitrogen, iron, and phosphorus, which is considered a direct mechanism of plant growth promotion (Figure 2).

#### 4.1.1. Nitrogen fixation

PGPB may prevent nutrient loss by fixing soil nutrients to increase the concentration and availability of nutrients in the rhizosphere. Common bacteria with biological nitrogen fixation are rhizobia and nitrogen-fixing bacteria, such as *Rhizobia* spp., *Azospirillum brasilense*, *Burkholderia* spp., *Gluconacetobacter diazotrophicus*, and *Herbaspirillum seropedicae* have been reported to increase host plant biomass by fixing atmospheric nitrogen (Bhattacharjee et al., 2008). In non-leguminous plants, PGPB has nitrogen-fixing effects by inducing the formation of nodules and secretes a unique nitrogenase that converts atmospheric nitrogen into ammonia (Aasfar et al., 2021). Endophytic bacteria are inferior to rhizobia in nitrogen fixation, while endophytic diazotrophic are very good in this respect. It has been found that *G. diazotrophicus* co-exists with plants such as spruce and pine, helping the conifers to grow in soils with severe nitrogen deficiency (Carrell and Frank, 2014).

#### 4.1.2. Inorganic phosphorus solubilization

Although the content of phosphorus in soil is generally quite high, it occurs mostly in insoluble form and cannot be used by plants (Etesami, 2020). In addition, much of the soluble inorganic phosphorus used as fertilizer can form insoluble P-complexes with soil particles (Elhaissoufi et al., 2021). The limited bioavailability of phosphorus in soil, coupled with the importance of this element for plant growth, limits plant growth. Therefore, phosphate solubilizing PGPB can promote plant growth by the solubilization and mineralization of phosphorus, which is achieved by the release of phosphate from inorganic phosphorus and organic phosphorus by organic acids such as acetate, succinate, citrate and gluconate, or by phosphatases (Rebi et al., 2022). Endophytic bacteria can use many mechanisms such as acidification, chelation, ion exchange, and production of organic acids and phosphatases to increase the availability of soil phosphorus (Walia et al., 2017). Recent studies have proposed a new mechanism of phosphorus solubilization, which 2, 3-dimethyl fumaric acid, gluconic acid and butylamine secreted by *Pseudomonas prosekii* can dissolve phosphate by providing H^+^ ions and organic anions (Yu et al., 2019). The phosphate-solubilizing bacterium *Burkholderia cepacia* can secrete gluconic, formic acids and an unknown acid, which might be an important mechanism for phosphate solubilization (Pande et al., 2019).

#### 4.1.3. Siderophore

Like phosphorus, a great amount of insoluble iron in soil is not available for plants. In iron-deficient conditions, bacteria can effectively bind iron through two strategies, including releasing protons and organic acids to reduce soil pH, or secreting iron-chelating complexes (siderophore) that bind ferric ions. Plants can obtain iron from siderophore through root chelation degradation or ligand exchange (Albelda-Berenguer et al., 2019). PGPB prevents the proliferation of plant pathogens by secreting siderophores with an extremely high affinity for ferric iron, and the mechanism is that these siderophores bind tightly to most of the Fe^3+^ present in the rhizosphere and absorb the bound iron into the PGPB or host plant, preventing any fungal and bacterial pathogens from obtaining enough iron for their growth (Olanrewaju et al., 2017). As a result, pathogens are unable to reproduce due to iron deficiency, causing them to lose their ability to act as pathogens. The effectiveness of this biological control approach is based on the fact that the siderophores secreted by PGPB have a much higher affinity for iron than fungal siderophores (usually by many orders of magnitude) (Olanrewaju et al., 2017). Siderophores as iron chelators have been shown to promote plant growth significantly in many studies. When iron is not available, PGPB *G. diazotrophicus* and *A. brasilense* could produce hydroxamate and catechol type siderophores, respectively, to chelate iron and promote host absorption (Delaporte-Quintana et al., 2020). Another study has shown that some bacteria secrete growth-inhibitory siderophores that alter the interaction between the microbiome and pathogens. Rhizosphere microbiome members secreting growth-inhibiting siderophores could inhibit pathogens *in vitro* as well as in natural and greenhouse soils. Conversely, rhizosphere microbiome members with growth-promotive siderophores are often at a disadvantage in the competition and easily facilitate plant infection by the pathogen (Gu et al., 2020). This is one of the reasons why plants inoculated with PGPB show less of a growth promotion effect than expected.

### 4.2. Production and regulation of plant hormones

The physiological activities of most plants are regulated by one or more plant hormones. In addition to plants, many beneficial microbes can also synthesize plant hormones (Figure 2) (Khan et al., 2020). PGPB can promote nutrient uptake and metabolism of host plants by producing plant hormones (Stegelmeier et al., 2022). Studies have found that PGPB can secrete plant hormones, resulting in facilitated seed germination, accelerated root growth, changed root morphology, and increased root biomass (Olanrewaju et al., 2017). At present, many studies found that PGPB *Azospirillum*, *Aeromonas*, *Azotobacter*, *Bacillus*, *Paenibacillus*, *Burkholderia*, *Enterobacter*, *Pantoea*, *Pseudomonas* and *Rhizobium* are able to secrete plant hormones (Khabbaz et al., 2019). Plant hormones generally include abscisic acid, cytokinin, ethylene, gibberellin and indole-3-acetic acid (IAA); among them, IAA and ethylene are the two classical phytohormones in the plant-bacteria interaction (Stegelmeier et al., 2022).

#### 4.2.1. IAA regulation

As a major plant hormone, IAA is involved in plant development and physiological processes, including intercellular signal transduction, regulation of plant growth, and induction of plant defense system (Tian et al., 2018). It can also facilitate the formation of lateral and adventive roots, affect photosynthesis and biosynthesis of metabolites, and mediate resistance to stress conditions (Li et al., 2021). IAA produced by PGPB and the plant’s endogenous IAA stimulate auxin signal transduction pathways, including various auxin response factors, thus resulting in stimulation of cell growth and proliferation (Ilangumaran and Smith, 2017).

IAA synthesized by bacteria may be involved in plant-bacteria interaction at different levels, especially in plant growth and nodule formation (Lobo et al., 2023). IAA produced by PGPB can either stimulate root development when the plant inherent IAA is suboptimal, or inhibit root growth when the concentration of IAA is already optimal (Munir et al., 2022). In addition, a plant’s endogenous IAA levels can also determine whether bacterial IAA promotes or inhibits plant growth, since bacterial IAA production generally favors plants with low endogenous IAA levels. Overall, bacterial IAA increases plant’s root surface area and length, thus providing the plant more access to soil nutrients (Yadav et al., 2022). In addition, bacterial IAA can loosen the plant cell walls, thus promoting an increase in the amount of root exudation that provide additional nutrients to support the growth of rhizosphere bacteria (Ahmad et al., 2022).

#### 4.2.2. ACC deaminase

Ethylene is an important plant hormone involved in different developmental and physiological processes such as aging, shedding, and pathogen defense signals (Bayanati et al., 2021). Some PGPB possess 1-aminocyclopropane-1-carboxylate (ACC) deaminase activity, which hydrolyzes the ethylene precursor, ACC, into α-ketobutyric acid and ammonia (Yadav et al., 2022). Therefore, hydrolysis of ACC can alleviate ethylene stress on plants and improve plant growth under stress conditions. Many PGPB can utilize ACC as a sole nitrogen source, indicating that these strains contain ACC deaminase (del Carmen Orozco-Mosqueda et al., 2020). So far, ACC deaminase in PGPB has proven to be a very effective mechanism to counter stress conditions in plants. However, these studies are limited to greenhouses and growing chambers.

Studies have shown that IAA promotes the transcription of genes encoding ACC synthase, thereby increasing ACC concentration and ultimately leading to ethylene accumulation (Glick, 2014). After environmental stress, PGPB containing ACC deaminase hydrolyzes ACC and reduces the ethylene level of plants, thus reducing the stress response of plants. In the absence of ACC deaminase-producing bacteria, ethylene restricts cell growth and proliferation by restricting auxin response factor transcription and stimulating IAA to produce additional ethylene (Khabbaz et al., 2019). Thus, in the presence of ACC deaminase, auxin response factor transcription is not inhibited, and IAA can stimulate cell growth and proliferation without causing ethylene accumulation. Therefore, ACC deaminase can not only reduce the inhibitory effect of ethylene on plant growth, but also enable IAA to promote plant growth to the maximum extent in the presence or absence of plant stress.

#### 4.2.3. Cytokinin and gibberellin

In addition to IAA, many PGPB also produce cytokinin and gibberellin (Kumar et al., 2020). Cytokinin influences not only many aspects of plant growth, development and physiology, including cell division, chloroplast differentiation and delay of senescence, but also numerous plant-biotic interactions (Akhtar et al., 2019). It is worth noting that cytokinins are produced by both pathogenic and beneficial microbes and improve the resistance of plants against pathogen infections. Transgenic plants that overproduce cytokinins, especially during abiotic stress, can significantly mitigate the harmful effects of stress (Rivero et al., 2007). PGPB *R. leguminosarum*, *Bacillus*, and *Pseudomonas* can produce cytokinin, promote cell division and growth, enhance the absorption efficiency of nutrients, and thus as a result improve plant yield (Olanrewaju et al., 2017). In terms of biotic stress, studies have confirmed that cytokinin can initiate plant responses to trauma and insect pests by activating the expression of trauma-inducing genes and by inducing the increase of compounds against insect (Giron et al., 2013), which indicates that the physiological and metabolic signals of cytokinin may interfere on anti-herbivore defense in foliage (Akhtar et al., 2019).

Gibberellin is a plant growth regulator mainly involved in seed germination, leaf and stem growth, flowering, fruit formation and plant senescence (Desta and Amare, 2021). Bacteria that have been shown to produce a variety of gibberellins include: *Acetobacter diazotrophicus*, *H. seropedicae*, *A. lipoferum*, *Rhizobium phaseoli*, *Enterococcus faecium*, *Sphingomonas*, etc. (Ali et al., 2017). Compared with the research on cytokinins, relatively limited research has been done on the interactions among gibberellins and plants and microbes. In most cases, gibberellin produced by PGPB also exhibits other mechanisms that promote plant growth, and the phenotypic changes of different plant varieties are related to changes in plant growth hormone biosynthesis (Kurepin et al., 2015).

### 4.3. Inhibition of plant pathogens

Endophytic bacteria indirectly promote the growth of host plants by inhibiting plant pathogens and pests with the production of antagonistic compounds such as antibiotics, toxins, siderophores, lytic enzymes, and antimicrobial volatile organic compounds (Figure 2) (Elnahal et al., 2022). Among them, *Actinobacteria*, *Bacillus*, *Enterobacter*, *Paenibacillus*, *Pseudomonas* and other bacteria are common genera with antibacterial activity against plant pathogens (Afzal et al., 2019). Some of the enzymes produced by PGPB, including chitinase, cellulase, glucanase, protease and lipase, can lyse a portion of the cell walls of pathogens (Ghosh et al., 2021). PGPB that secrete one or more of these enzymes have been shown to have biocontrol activities against a wide range of competitor pathogens (Selari et al., 2023). Naama-Amar et al. (2022) reported that a candidate biocontrol agent *Frateuria defendens* could inhibit *Spiroplasma melliferum* growth by secreting antimicrobial metabolites (Naama-Amar et al., 2022). A plant growth-promoting endophytic bacterium *Pseudomonas protegens* MP12 can produce important antifungal compounds (2, 4-diacetylphloroglucinol, pyoluteorin and pyrrolnitrin), and exhibits inhibitory effects on the mycelial growth of phytopathogens such as *Botrytis cinerea*, *Alternaria alternata*, *Aspergillus niger*, *Penicillium expansum* and *Neofusicoccum parvum* (Andreolli et al., 2019). *Bacillus* produced antibiotic lipopeptides, such as iturin, bacillomycin, bacilysin, fengycin, surfactin, and zwittermycin, which have broad spectrum of action against pathogens (Saravanakumar et al., 2019).

PGPB can also activate plant-induced systemic resistance (ISR) against a broad spectrum of pathogens (Datta et al., 2022). ISR is phenotypically similar to systemic acquired resistance (SAR) that occurs when plants activate their defense mechanisms in response to pathogen infection (Meena et al., 2020). The prime function of ISR is to activate plant defense mechanisms and protect unexposed parts of plants against pathogenic microbe and herbivorous insect invasions (Sardar et al., 2021). PGPB-mediated ISR depones on jasmonic acid and ethylene signaling pathways in plants (Abid and Karim, 2021). These hormones stimulate the host plant’s defense responses to a range of pathogens without the need for PGPB to interact directly with pathogens. In addition to involvement in jasmonate and ethylene signaling pathways, ISR also plays a crucial role in host resistance by enhancing the activity of pattern recognition receptors through cellular or hormonal defenses (Hossain and Chung, 2019).

PGPB can also emit volatile organic compounds (VOCs) as biocontrol factors or deterrents against pathogens (Choub et al., 2022). Recently, many studies have reported the production of VOCs secreted by PGPB that disrupted cell membrane integrity, spore germination and mycelial growth of plant pathogenic fungi and other competing bacteria species (Chatterjee and Niinemets, 2022). Recent advances in analytical science such as the headspace solid-phase microextraction/gas chromatography–mass spectrometry (HS-SPME/GC–MS), have been used for extraction, identification, and characterization of VOCs emitted by PGPB and other bacterial species (Drabińska et al., 2022; Tabbal et al., 2022; Wu et al., 2022). Additionally, some VOCs can repress the expression of virulence traits involved in host colonization, such as motility, root colonization, and biofilm formation, thus, for example, effectively controlling tomato wilt (Raza et al., 2016). In summary, VOCs produced by PGPB help plants cope with abiotic and biotic stresses by inducing systemic resistance or inhibiting the growth of a wide range of pathogen.

In addition to producing substances that harm or inhibit plant pathogens, some PGPB may compete for nutrients or niches with pathogens (Selari et al., 2023). In fact, it is widely believed that this competitiveness of PGPB works together with other biocontrol mechanisms to repress the growth of plant pathogens (Olanrewaju et al., 2017). It is important to note that the effectiveness of PGPB depends on their versatility and adaptation to new niches as well as their ability to colonize and compete with other members of plant microbiome (Rilling et al., 2019). Root exudates contain chemo-attractants like amino acids, organic acids and specific sugars that are good carbon sources for PGPB activity and competitive colonization (Feng et al., 2021). Additionally, the mutual signal exchange between plants and microsymbionts, along with flagellar motility, further enhances the affinity of PGPB in root surface (Raina et al., 2019).

### 4.4. Osmoregulation

In addition to beneficial traits such as IAA production, nitrogen fixation and phosphate dissolution, PGPB can accumulate osmotic adjustment substances under stressful conditions (Figure 2) (Feng et al., 2023). Under drought and salt stress conditions, plant cells can improve the water activity of cells and maintain normal metabolic activities by accumulating compatible solutes (soluble sugars, polyols, glycine betaine, proline, free amino acids) (Ali et al., 2022). Under abiotic stress, one of the most significant changes is the accumulation of plant proline that can improve the activity of various enzymes, stabilize intracellular pH and remove reactive oxygen species, thereby maintaining antioxidant activity (Abdelaal et al., 2021). Many PGPB can induce plant proline synthesis under stress conditions, which helps to maintain cell osmotic pressure and improve plant salt tolerance (Khabbaz et al., 2019).

Trehalose, a non-reducing disaccharide, can act as a protective agent to alleviate a variety of environmental stresses, including drought, salinity and extreme temperatures (Greco et al., 2021). When cells are dehydrated, trehalose can form a gel phase to bind with surrounding macromolecules and membranes thereby replacing water loss and decreasing the damage to cells (del Carmen Orozco-Mosqueda et al., 2022). In addition, trehalose prevents the degradation, denaturation and aggregation of proteins under high and low temperature stress (Vinciguerra et al., 2022). Under stress conditions, trehalose and ACC deaminase synergistically protect crops from stress by stabilizing biological structures and lowering ethylene levels, respectively (del Carmen Orozco-Mosqueda et al., 2022). Compared with wild type *Rhizobium etli*, the number of nodules, nitrogenase activity and biomass of *Phaseolus vulgaris* increased when inoculated with *R. etli* overexpressing trehalose synthase, and the host plant inoculated with mutants recovered more strongly under drought stress (Suárez et al., 2008). Although it is also possible to overexpress trehalose genes in transgenic plants, it is much simpler to achieve the same goal through co-inoculation with PGPB. Moreover, since most strains have no specific host, therefore, the same strain can effectively help different plants mitigate stress.

## 5. Relationship between bacterial Type VI secretion system and plant growth promotion

Many bacteria have evolved specialized protein secretion systems to transport proteins out of the bacterial cells and even directly into the host target cells. Bacterial secretory systems are divided into at least nine classes (Tat system, type I-VII, and type IX) based on their structure, function, and specific effects, with each system transporting a specific subset of proteins, known as effectors (Green and Mecsas, 2016). Among them, Type VI Secretion System (T6SS) is a sophisticated nano-weapon to inject toxic effectors into eukaryotic or prokaryotic cells, and presents in nearly 25% of all Gram-negative bacteria, including many plant symbiotic bacteria (Salinero-Lanzarote et al., 2019). Bacteria can use T6SS to manipulate and destroy eukaryotic cells and/or fight other bacteria to gain a dominant status in the ecological niche (Kim et al., 2020). Indeed, bacteria with T6SS appear to have a significant adaptive advantage in the microbial community. Bacteria containing T6SS synthesize both immune proteins and T6SS effectors to ensure against self-poisoning or targeting by sister-cells (Steele et al., 2017). Functional T6SS contributes to the virulence of the host, improves bacterial robustness, and enhances environmental adaptation through bacterial competition (Russell et al., 2014; Kim et al., 2020). Although T6SS is ubiquitous, its general mechanism and physiological role are still not fully understood. To date, the studies related to T6SS and plant growth promotion characteristics are very few and mainly focus on biological control agents, antagonism, biofilm formation and environmental adaptability (Bernal et al., 2018).

### 5.1. Biofilm formation

Biofilms are structurally complex microbial communities attached to living or abiotic surfaces and surrounded by complex extracellular polymers. Microbial colonization of plant roots can be promoted by the formation of biofilms (Niu et al., 2020). Gallique et al. (2017) reported that mutations in three T6SS-related genes (*hcp1*, *hcp2*, or *hcp3*) of *Pseudomonas fluorescens* MFE01 did not reduce biofilm formation, but these three Hcp proteins are essential for the formation of mature biofilm structures. However, in another similar study, these three T6SS-related gene mutants of *P. aeruginosa* PAO1 had no impact on biofilm formation or environmental adaptation ability, but swarming motility was reduced, which is another important aspect of biofilm formation ability, suggesting that T6SS is associated with biofilm formation but not environmental adaptation (Chen et al., 2020). The T6SS gene cluster present in the *Acidovorax citrulli* was confirmed to be involved in multiple biological processes, including colonization, competition and biofilm formation (Fei et al., 2022).

### 5.2. Antibacterial activity

Evidence is also emerging that T6SSs could contribute to inter-bacterial competition. Interestingly, many PGPB harbor one or more T6SS, however, the function of T6SS in PGPB is poorly understand. In a previous study, it was found that the T6SS of PGPB *Azospirillum brasilense* provides antibacterial activities against a number of plant pathogens *in vitro*, and might confer T6SS-dependent bio-control protection to microalgae and plants against bacterial pathogens (Cassan et al., 2021). However, Lin et al. (2018) found instead of being an antihost or antibacterial weapon of the bacterium, the T6SS in *Azorhizobium caulinodans* ORS571 seems to participate specifically in symbiosis by increasing its symbiotic competitiveness.

### 5.3. Colonization

Most organisms with T6SS are not pathogenic and are found in diverse environments as symbionts. Although the secretory system was thought to be involved in the interaction between PGPB and plants, only recently research has been functionally dissected in nitrogen-fixing *Azoarcus olearius*, in which T6SS positively affected plant colonization efficiency (Jiang et al., 2019). The T6SS effector Hcp1 up-regulated in rice inoculated with a well-known growth-promoting rhizobacteria *Herbaspirillum rubrisubalbicans*, suggesting that T6SS plays a role in rice colonization (Valdameri et al., 2017). Similarly, the T6SS mutant of PGPB *Enterobacter* sp. J49 showed a significant decrease in the epiphytic and endophytic colonization, indicating that although T6SS is not essential, it may participate in bacterial colonization (Lucero et al., 2022). Salinero-Lanzarote et al. (2019) described the growth-promoting trait of T6SS in *Rhizobium etli* Mim1, which can effectively increase nodule size in legumes, suggesting that T6SS plays an active role in the *Rhizobium*-legumes symbiosis. The knockout mutant of T6SS gene cluster in rice endophyte *Kosakonia* showed a decreased ability to colonize the rhizoplane and endosphere, suggesting that T6SS is involved in the colonization process of plant-bacteria interaction (Mosquito et al., 2020). Another plant-associated microbe, *P. taiwanensis*, has been shown to use its T6SS to mediate colonization resistance against bacterial plant pathogens through T6SS-mediated secretion of pyoverdine, an iron chelator (Chen et al., 2016). These results suggest that there may be a relationship between T6SS and plant growth promoting properties.

## 6. Conclusion and future prospects

Soil salinity is expected to increase dramatically in the coming years and will hinder agricultural production. Various conventional reclamation methods of saline-alkali land are found to be unsustainable and economically difficult to achieve. The use of PGPB to inoculate plants has become an important method to alleviate soil salt stress and improve crop yield, but better results have only been obtained in laboratory or greenhouse conditions, not in the field. To effectively utilize PGPB in salinized agroecosystems, a bottom-up approach is necessary, starting with the screening of halotolerant PGPB strains and careful formulation design for field application. Additionally, more molecular studies of plant-microbe interactions are needed to better understand the mechanisms involved in inducing systemic tolerance and performing rhizosphere engineering under salt stress. Studying the metabolic and genetic behavior of halotolerant PGPB is also important to understand how they work and adapt to high salt environments. This will provide a reference for the development of reliable biological inoculants in saline soils.

Although PGPB has various mechanisms to improve plant growth and biological control against plant pathogens under saline conditions, there are still many constraints to its wide application in different agro-ecosystems. Our understanding of the molecular mechanism by which halotolerant PGPB alleviates salt stress is also in its infancy, but it offers a sustainable way to increase the productivity of saline soils and contribute to the goals of food security and controlling desertification of agro-ecosystems. Improving our understanding of the mechanism of PGPB regulating plant stress resistance and restricting bacterial activity under stress conditions can enhance the effectiveness of using bacterial inoculants.

## Author contributions

MP wrote the initial draft and made figure. MP and ZJ edited the manuscript. FZ and ZW helped in collection and review of literature. All authors contributed to the article and approved the submitted version.

## Funding

This study was financially supported by the National Natural Science Foundation of China (No. 32200094), the Natural Science Foundation of Hubei Province (2022CFB674), and the Open Fund of Hubei Key Laboratory of Biological Resources Protection and Utilization (Hubei Minzu University) (No. PT012201).

## Conflict of interest

The authors declare that the research was conducted in the absence of any commercial or financial relationships that could be construed as a potential conflict of interest.

## Publisher’s note

All claims expressed in this article are solely those of the authors and do not necessarily represent those of their affiliated organizations, or those of the publisher, the editors and the reviewers. Any product that may be evaluated in this article, or claim that may be made by its manufacturer, is not guaranteed or endorsed by the publisher.

## Figures and Tables

**Table:** 

PGPB	Plant growth-promoting bacteria
PGPR	Plant growth promoting rhizosphere
IAA	Indole-3-acetic acid
ACC	1-aminocyclopropane-1-carboxylate
ISR	Induced systemic resistance
VOCs	Volatile organic compounds
HS-SPME/GC–MS	Headspace solid-phase microextraction/gas chromatography–mass spectrometry
T6SS	Type VI Secretion System
APX	Ascorbate peroxidase
SOD	Superoxide dismutase
CAT	Catalase
SOS	Salt overly sensitive
ETH	Ethylene
HKT	High-affinity potassium transporter
SAM	S-adenosylmethionine
CK	Cytokinin
